# Diabetes and conversational agents: the AIDA project case study

**DOI:** 10.1007/s44163-021-00005-1

**Published:** 2021-09-22

**Authors:** Francesca Alloatti, Alessio Bosca, Luigi Di Caro, Fabrizio Pieraccini

**Affiliations:** 1 H-FARM Innovation, Turin, Italy; 2 Department of Computer Science, University of Turin, Turin, Italy; 3 Novo Nordisk Spa, Rome, Italy

## Abstract

One of the key aspects in the process of caring for people with diabetes is Therapeutic Education (TE). TE is a teaching process for training patients so that they can self-manage their care plan. Alongside traditional methods of providing educational content, there are now alternative forms of delivery thanks to the implementation of advanced Information Technologies systems such as conversational agents (CAs). In this context, we present the AIDA project: an ensemble of two different CAs intended to provide a TE tool for people with diabetes. The Artificial Intelligence Diabetes Assistant (AIDA) consists of a text-based chatbot and a speech-based dialog system. Their content has been created and validated by a scientific board. AIDA Chatbot—the text-based agent—provides a broad spectrum of information about diabetes, while AIDA Cookbot—the voice-based agent—presents recipes compliant with a diabetic patient’s diet. We provide a thorough description of the development process for both agents, the technology employed and their usage by the general public. AIDA Chatbot and AIDA Cookbot are freely available and they represent the first example of conversational agents in Italian to support diabetes patients, clinicians and caregivers.

**Supplementary Information:**

The online version contains supplementary material available at 10.1007/s44163-021-00005-1.

## Introduction

Natural Language Processing has significantly improved over the past years, thanks to the development of new and powerful Artificial Intelligence algorithms and computational methodologies. Results of this improvement include the growth of conversational technologies in specific fields, such as healthcare [1, 2]. However, healthcare is a broad domain and not all conditions or situations are equally represented in the Conversational AI landscape. Diabetes is one of many topics that has been only partially tackled by computer science.

The standard treatment for diabetes poses several goals and challenges [3]. For instance, the preservation of adequate blood glucose values, expressed by the levels of glycated hemoglobin, has been associated with a lower risk of disease related complications compared to progressively increasing blood glucose levels [4–6]. One of the goals of treatment is the prevention of complications. However, the employment of numerous—and effective—pharmacological therapeutic strategies cannot ignore the factor of patient satisfaction: their quality of life, both objectively measured and subjectively perceived, is an extremely important component in the process of care.

Unfortunately, despite the availability of innovative drugs and medical devices, a high proportion of people with both type 1 and type 2 diabetes fail to reach the desired therapeutic targets [7]. This issue questions the standard approach to patient management and it has a significant economic impact on health systems too [8, 9]. In this context, one of the key aspects in the process of caring for people with diabetes is Therapeutic Education (TE) [10]. TE is a teaching process that aims to train patients to self-manage the aspects of their life related to the illness [11]. Autonomy in care management allows patients to reach a new level of well-being; it is also a more flexible way of approaching the process, as it can be adapted as the disease evolves, or to changes in people’s way of life. Such educational interventions are always aimed at improving patients’ knowledge and practical ability to manage their care plan. The final goal is to modify their behaviors until a balance is obtained between the patients’ needs and the constraints of the disease. The goal of TE is therefore to make patients with diabetes as conscious and autonomous as possible in the management of glycemic measurements, in the choice of the most appropriate therapy (for example the insulin dose) and in the management of any acute complications related to the disease.

Alongside traditional methods of providing educational content, patients and caregivers can now enjoy new alternative forms of information transmission. Nowadays, people with diabetes may use technological support such as websites, apps, or digital assistants. Artificial Intelligence (AI) applications can provide assistance in a personalized way and can amplify the machine’s ability to connect different medical sources, thus providing patients with new tools to obtain and manage information regarding their condition [12–14]. In the healthcare domain, one of the most promising benefits of AI technologies is their ability to create new forms of dialogue systems. Those systems, also known as conversational agents (CAs), are equipped with the ability to understand natural language and manage complex information exchange [15–17]. TE can now be delivered through the tool of human-machine dialogue which brings a series of benefits: patients can obtain information autonomously, simply by interacting with a chat interface; CAs are usually available at every hour, while a doctor may not always be available for a rapid consultation; moreover, patients who interact with a machine may be more inclined to ask about sensitive topics that they do not feel comfortable addressing with a person [18, 19].

In this article we present the Artificial Intelligence Diabetes Assistant (AIDA) project, which includes two CAs designed and built with the explicit goal of providing patients with an innovative tool to improve their access to therapeutic information. Specifically, our contribution consists it:

- Highlighting the design process upon which AIDAs are built. Especially in the healthcare domain, CAs should be built with particular attention towards their potential users. We employed established techniques in the human–computer interaction domain to inform the design;
- Outlining the architecture that empowers the system’s reasoning. AIDAs exploit both machine learning capabilities and a rule-based engine. We believe that the balance of these two modules constitutes the best approach for this kind of CAs and their description is a unique insight into industrial technologies for conversational AI;
- Providing the system’s usage data. AIDAs were made available to the public during the year 2020. Our first goal was to ascertain the usability of such tools within the diabetic population of Italy and their reaction to CA technology. We present usage data gathered *in the wild* and provide insights into the behavior of the users when interacting with CAs for TE.

The case study offers a novel perspective at the crossroad of two disciplines: therapeutic education and conversational AI. Our goal is to provide the scientific community with information about industrial CAs, which are often developed solely on private company premises and the features of which are usually not revealed to the public.

Artificial Intelligence Diabetes Assistant (AIDA) is a chatbot delivered via a written channel as well as a voice-based assistant conveyed via an Alexa Skill.^1^ For the text-based CA, we refer to it as AIDA Chatbot; in the second case, as AIDA Cookbot. They are AI-based conversational systems devised by the pharmaceutical company Novo Nordisk and created by computational linguists from the NLP company CELI, with the support of a scientific board. Both AIDAs use the Italian language and are mainly directed to Italian speakers.

The voice-based agent focuses on the diet regime theme and provides recipes for users. It was deemed that a speech system would be more engaging and effective in delivering this kind of content. The text-based agent, on the other hand, has a broader scope: it answers numerous questions about diabetes and offers help for people with type 1 and type 2 diabetes, caregivers and general practitioners. Since its goal is to answer questions, rather than engaging the user, the written channel was deemed to be more appropriate.

The goal for both is to generate awareness about diabetes among the general public and help those who have already been diagnosed to easily obtain information and advice about everyday questions. Especially during the COVID-19 pandemic, the tool would help people obtain secure information in an autonomous way, as well as help clinicians to focus on more pressing issues while delegating simpler educational tasks to a machine [20, 21].

The article is structured as follows: in Sect. 2 we provide some background information by analysing related work and the differences between previous studies and the AIDA project. In Sects. 3 and 4 we describe the start of the project: specifically, how the initial knowledge base for both the chatbot and the voice-based assistant was created, its adherence to scientific principles, and the User Experience precepts that guided the whole project. AIDA is built upon proprietary software integrated with external endpoints when appropriate. Its architecture is described in Sect. 5. In Sect. 6 the authors describe the technology behind the Conversational Agent: the components that allow the reasoning to take place and its relevant infrastructural details. Once the CA was built, it was made available to the public by means of a dedicated landing page, a third-party chat system (Telegram) and through Amazon Alexa. Our goal was to provide patients and caregivers with ready to use tools as soon as possible during 2020. Section 7 describes the release of the system in production and provides data about its usage and performance. The article ends with a Conclusion and prospects for the future path of AIDA and Therapeutic Education Technologies.

## Related work

Conversational interfaces allow humans to interact with devices using “natural language”, that is, the set of words and structures that humans use daily to express themselves. Instead of communicating with the machine through a formal system that the computer can understand (like a programming language), the user can speak or write freely, delegating the interpretation of the message to mechanical components [22]. The application of CAs in the healthcare sector is a relatively recent phenomenon, in fact most of the studies have been carried out starting from 2010 [23, 24]. Since then, however, there has been a steep increase in research and publications [25, 26]: several experiments have introduced CAs that help patients and doctors manage the treatment process more effectively.

With regard to patients, CAs are tools that provide health information and help patients manage their drug therapy or comply with physicians’ instructions [27, 28]. These interfaces can also help doctors, for example by conducting medical history interviews, thus relieving the amount of repetitive and impersonal work [29]. The various conversational systems can be categorised according to certain characteristics [23]: the type of input they accept (written, spoken, or both); whether the action is agent-driven or user-driven (i.e. whether the system asks questions to guide the user or vice versa); on which platform they are hosted (a dedicated app, a web interface, a bot on a messaging application, etc...); their purpose (chat-oriented or task-oriented). It is useful to note that most of the interfaces in the healthcare field are currently task-oriented agents—that is, they help patients, doctors or caregivers carry out a task—rather than chat-oriented agents whose only goal is to perform casual conversation.

### Conversational agents and diabetes

In the domain of diabetes, some studies on educational tools to facilitate pathology self-management have been carried out [30–33]. However, most of them concern either type 1 or type 2 diabetes patients, while our focus is on both: as will be detailed in Sect. 3, one of the requirements was to design a system that would cover questions from both types of patients. Many studies either concentrate on the information needs of patients [34, 35], or the building of an AI-based agent [36–38], rarely both. Although some studies focus on the theme of recipe recommendation [39, 40], the recipes are not specific to people with diabetes or they are not delivered by means of a voice-based agent.

Other studies concern therapeutic education for diabetic patients but are not CAs [41, 42], or they are CAs but not specifically aimed at diabetes patients [43]. The first premise of our work was to provide patients with a conversational tool. One of the closest studies to ours is by Stephens et al. [38]. They illustrate Tess, a support tool for obese children in a pre-diabetic stage. Tess is also equipped with a pass-to-human feature that appears to be seamless, without the users being aware of the switch between machine and human. AIDA does not involve a pass-to-human intervention at any of its conversation stages; the only human intervention comes into play during the Validation task. This decision has the clear purpose of experimenting with the agent only, measuring its performance only afterwards and not making any human corrections mid-interaction.

Wang and colleagues [35] describe the process of classifying users’ questions about diabetes. The questions were obtained by crawling *39 Health*, a popular health related website in China. The crawling approach differs from that of AIDA, where the KB was manually created by the scientific board, while it is more similar to the work by Crangle et al. [44]. Moreover, AIDA’s KB is in Italian, differentiating this work from previous ones [45]. By obtaining questions from a website, Wang and colleagues managed to capture from the very beginning all the different linguistic forms employed by real patients. However, the classification tasks alone would start from spurious data, slowing down the whole development process. Moreover, the linguistic features clinicians apply when talking to patients are specific and they should be applied when chatbots are conversing with patients [46]. Correct use of medical language has been proven to be essential to the positive outcome of the treatment [47] and a CA should use the same terminology that is used by clinicians. The DI@L-log agent described by Black et al. [8] even poses as a nurse. In our case, the scientific board thought that it would be unethical to imply that AIDA had the same capabilities as a human clinician.

### Multimedia systems

Some systems exploit multimedia content along text-based exchanges, such as voice, videos or images [37]. For instance, Emmi appears to be a ready-to-use web app that provides text and multimedia content [36]. However, it is not clear whether Emmi is a conversational assistant (since it answers questions via voice), or whether it consists of a Graphical User Interface enriched by audio and videos.

Frøisland and Årsand [34] propose an application made of two components. The first is a mobile app that allows users to take a picture of their meal; by doing this, patients create a diary of their own eating habits, thus making themselves more aware of their food consumption. The second component is a SMS communication protocol established between patients and their care provider. The process is fully managed by humans though, while in this context a CA could automatize the communication protocol.

Maharjan and colleagues [48] propose an Alexa-based technology to deliver information about nutritional values specifically for diabetic patients. This work is particularly interesting because it demonstrates the feasibility of such an intervention and therefore the scientific soundness of AIDA Cookbot. However, their Alexa skill is in English and aimed specifically at the Native American population. In our case, we refer to the Italian public, which has different eating habits. Moreover, their system recommends recipes but does not guide users in the actual preparation of the meal.

In general, CAs in the healthcare domain are a constant presence, as are studies aimed at enhancing Therapeutic Education for people with diabetes. The intersection between these two domains, however, has not yielded many results yet.

## User experience design of the conversational agent

In order to build agents compliant with the final users’ expectations, the design process takes into consideration requirements expressed by the main stakeholders of the project: patients and diabetologists. Patients are framed as the future main users of the systems, while diabetologists are the ones that, thanks to their medical expertise, will provide the KB (Knowledge Base, i.e. the content of the agents). Their needs and suggestions guided both the construction of the KB as well as the type of interaction that the agents would conduct with their users.

### Requirements gathering

The AIDA project began with a series of interviews. This methodology is widely used in the domain of human–computer interaction for CA, where qualitative interviews provide meaningful insights into the expectations and the mental model of future users [49–51].

The interviews had two purposes: first, to clarify the scenario within which the CA will have to be situated in terms of perception, expectations and desires; second, to find out what themes users would like to discuss with a CA [52]. The interviews were structured in the form of two interactive workshops. In the first workshop, the participants were eight diabetologists while the second workshop involved eight people with diabetes type 1 or 2.

Each group was balanced according to three criteria:

- *Gender and age*—in the first group there was a slighter predominance of males (M = 5, mean age = 40.4), while between patients, the majority identified as women (F = 6, mean age = 38.3).
- *Inclination to use digital tools*—the majority of clinicians declared they were inclined to rely on digital tools, either because they recognized they could empower patients’ independence (N = 3), or because they considered them to be more practical than paper-based systems (N = 3). The remaining two expressed more skeptical views. Between patients, the inclination correlated heavily with age: patients were comfortable with digital devices in the range 25–40 years (N = 3) and in the range 41–60 years old (N = 2); in the range 61–80 (N = 3) they were less comfortable.
- *Type of diabetes*—this criteria is only applicable to the second group. Older people had type 2 diabetes (N = 5), while younger ones type 1 (N = 3).

**Fig. 1:**
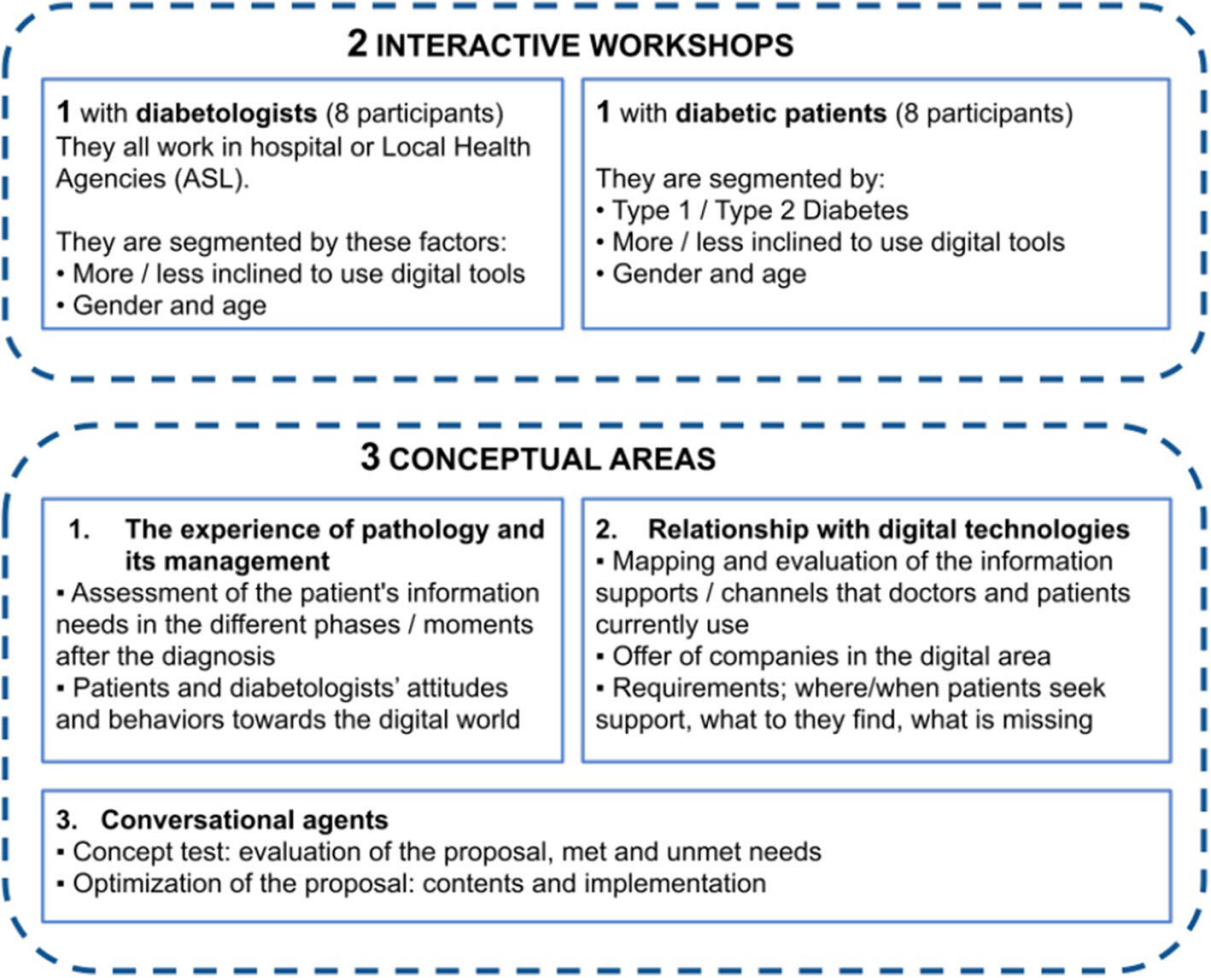
Participants and goals of the two interactive workshops

Figure 1 summarize the scope and features of the two workshops.

The workshops tackled three main conceptual areas that are relevant to both parties: (i) the experience of pathology and its management; (ii) doctors and patients’ relationship with digital technologies; (iii) thoughts about the proposal of a CA. Results were extracted by means of qualitative interviews.

In the first area, the two groups agreed on the need for patients to understand the pathology and all its consequences, so that they can absorb the necessary information to manage the pathology and control any possible outcome. During the time that follows the diagnosis, patients need to be reassured about the new challenges and doubts they are now facing. The questions that patients pose to their diabetologists relate to everyday aspects of their life: lifestyle advice, such as what kind of sport is permitted, how often, which food is better to eat and when to schedule one’s own meals. In this phase, patients feel insecure and often seek psychological support to help them accept the pathology and the social adjustments that are necessary. While on one side diabetologists need to inform persons in a reassuring but also firm way, on the other side patients need to resolve all their doubts, even minor ones, in order to accept their new reality.

Diabetologist: *the most frequent question I get at the beginning is “Will I ever be cured?”*

Patient: *why did it happen to me? Nobody in my family has diabetes...*

In the domain of digital technologies, both doctors and patients’ perceptions change according to their age group. While there is a general familiarity with technological instruments (some of which are an everyday necessity, such as the glucometer), younger generations feel more comfortable with them: they look for apps and IoT devices that can help them monitor their situation and share their data directly with their physician. In general, all groups use internet search to look for answers to their doubts, although younger patients profess to using only specialist sites because they do not trust any information that may come up from a Google search or in a Facebook group. All age divisions agree on the need to receive verified information through trustworthy channels that can be reached from various endpoints, without any discrepancies between a channel and the other.

Diabetologists, on the other hand, feel less comfortable with a plethora of digital devices, as they see potential privacy issues as well as an increased burden on their part to gather and analyse data from different sources. For them, ease of use, security in data management and re-use of existing applications is an essential requirement of a new technology.

Diabetologist: *it’s an issue of time and resources, most of the time the devices will not simplify my job, although they do help to monitor the patients*

Patient: *I look up special recipes on YouTube; [Internet] is ok, but you can find all sort of true and fake information there*

Finally, doctors and patients expressed their views about a CA that would provide information about diabetes. Both parties declared their need for a generalist tool, a system that would contain all sorts of information about pathology and the answers to all those lifestyle doubts that emerged before. Moreover, they wanted the agent to provide psychological support in the form of positive reinforcement and personalized motivational messages. They all agreed that a certain degree of customization would be preferable, even though that would imply sharing personal information.

Patient: *knowing how many carbs a certain food has would be helpful. For instance, I didn’t know that even tomatoes have carbs...*

The insights gained via the interview produced a detailed report of the requirements and expectations of both stakeholders.

### Design guidelines

The design guidelines were informed by the report produced after the workshops: the needs and suggestions that emerged during the interviews were used as *dos and don'ts* to create both CAs. To our knowledge, this is one of few cases where the design guidelines were informed by interviews and at the same time they were empirically applied to the construction of two different CAs [49, 53–55].

The first area indicated that the agents should be a reassuring presence, trustworthy and reliable from a medical point of view. Their internal knowledge should reflect that of the diabetologists and their tone of voice should be firm. Since many patients also need psychological support after the diagnosis, the agents should employ empathetic language and encourage them to reach out to family, friends and professional help if they express any discomfort.

The second area expressed various preferences in terms of devices, technologies and privacy. Apart from the soundness of the information that the agents would give, which was always considered as a paramount guideline, the workshops instructed the design of an agent that could be used from any device and that did not collect any personal data about the user. Ease of use and availability was one of the requirements suggested by the clinicians; by deploying the agents on a website, a free and popular messaging app and one of the most known voice-based platforms in Italy, we met those needs. The opportunity of both a text-based and a voice-based interaction also met the demands of the different age groups, that may find one agent or the other easier to use according to their familiarity with new technologies. Even though patients desired personalized interactions with the agent by sharing some of their data, it was deemed best not to collect any personal information about the users in order to comply with pharmaceutical regulations. Therefore both CA are agnostic to users and the interactions do not take into account any external information.

Ease of use piloted AIDA Chatbot towards user-driven interaction, where the user would be free to ask even impromptu questions without the need to engage in a complex and guided dialogue session. AIDA Cookbot, on the other hand, was created as agent-driven interaction because of the different communication channel it would exploit: via voice, it is important that the agent instruct users on what to say next in order to benefit from the CA’s content.

## Construction of the knowledge base

The results of the interview stated the principles that were later followed in the design of the CA’s KB. The KB was built during a co-design workshop, where a scientific board drafted the content guided by three facilitators and two computational linguists. Unlike previous work [33, 35], AIDAs’ KB was built manually, in order to assure that both questions and answers were scientifically sound as well as linguistically correct.

The scientific board was composed of four diabetologists, four specialists from the pharmaceutical company, and a psychologist. The presence of these different figures assured medical accuracy and adherence to pharmaceutical regulations. The psychologist in the group advised the board on the themes that were important to address according to the interviews as well as the tone that was appropriate to use while responding to certain doubts, some more delicate than others [56].

The scientific board agreed that the most important issue was the personal safety of the user: no patient or caregiver should ever find herself in a dangerous situation because of the information provided by the agents [57]. Moreover, no data exchange with other systems or request for personal data from the patient should be allowed, in order to meet the diabetologists’ concerns for privacy. AIDA would not be a predictive tool, therefore it could not be a medical device. As such, it could never provide treatment instructions or information about medication. It should be clearly stated that the agents are not a substitute for medical advice.

While the text-based AIDA would answer questions related to diabetes from a broad set of themes, AIDA Cookbot focusses on the food theme by supplying recipes that are compliant with a diabetic regime. Therefore, the board was tasked with the creation of the Q&A corpus, as well as with the recipe corpus.

### AIDA Chatbot’s KB

During the workshop, the multidisciplinary team selected four main themes that were deemed particularly important:

- Diagnosis—all the information about symptoms, cures and statistics on diabetes;
- Lifestyle—this section encompasses subjects such as sport, habits and all the services that are available for people with diabetes: fiscal exemption, measures for children in school, etc.
- Diet—one of the major issues for patients is the kind and quantity of food they are allowed to eat. This section aims to debunk false preconceptions about the strict connection between diabetes and diet habits, and supply sound information about correct food consumption.
- Prevention and complications—information about the prevention of diabetes as well as the possible consequences of a disease that, if not properly managed, can lead to severe consequences.

**Fig. 2:**
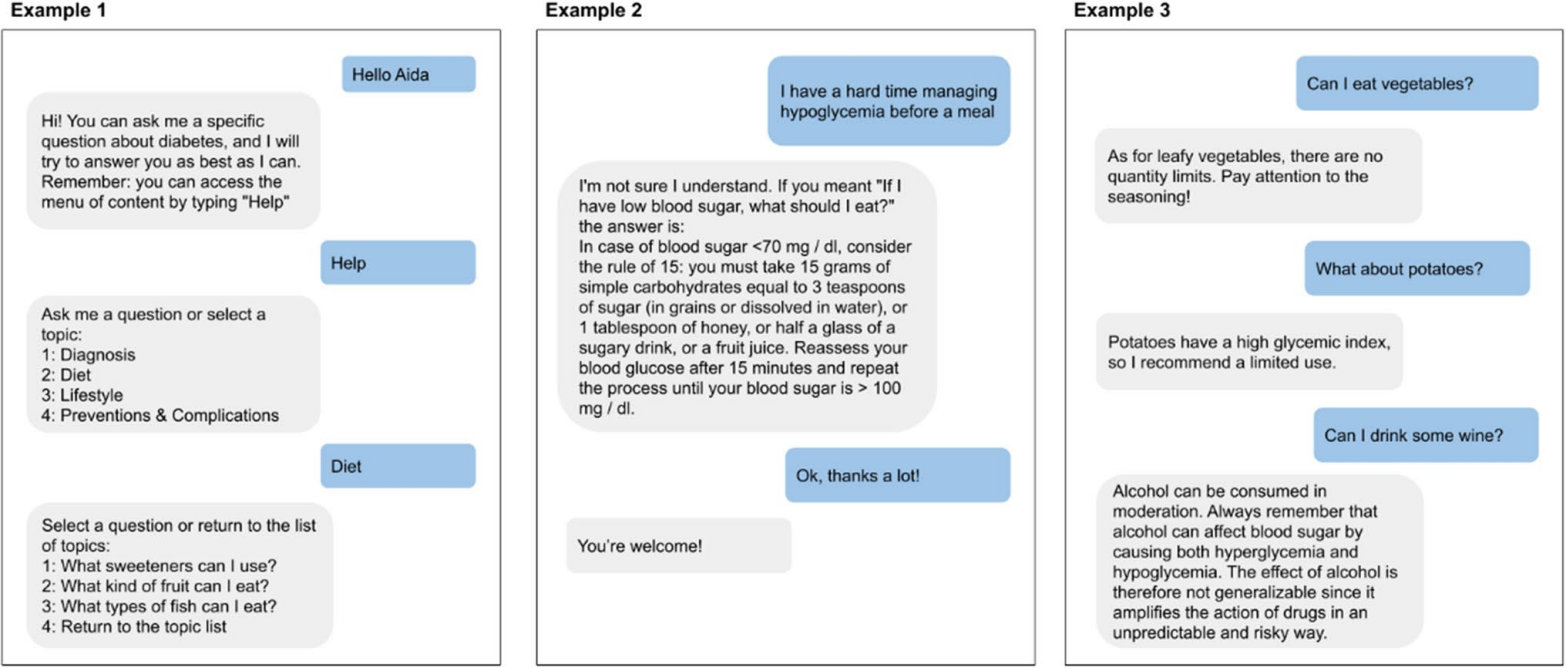
Some example dialogues. In Example 1, the user decides to consult AIDA by interacting with the content menu. In Example 2, the user asks a very specific question and AIDA tries to match it to the closest one in its KB. In Example 3, the user asks a series of questions related to the same topic. AIDA keeps track of the current topic in order to answer appropriately. The dialogues, originally in Italian, have been translated into English for the purpose of this article

For each of these themes, the scientific board drafted several questions that the user may ask and wrote the answers. The psychologist revised those answers in order to make them empathetic, respectful of the emotions of the patients. Table 1 presents all the main themes with their sub-themes, the number of questions each one contains and a couple of examples for each category. Figure 2 presents some example dialogues.

**Table 1:** AIDA Chatbot’s knowledge base is composed of 170 questions

Main themes and sub-themes	Questions	Examples
**Diagnosis**		
Incidence of diabetes	11	Is diabetes only due to genetic predisposition? Is there a risk of dying from diabetes?
Treatments for diabetes	10	Are insulin injections painful? How do glycosuric drugs work?
Symptoms of diabetes	16	My legs get swollen, is it because of diabetes? What are the symptoms of diabetes?
General information	25	Is there any danger related to Covid-19 for people with diabetes? What is more important, blood glucose or blood sugar?
**Lifestyle**		
Daily habits	20	How can diabetes affect my social life? How do I tell my loved ones that I have diabetes?
Services for diabetics	4	I have diabetes and I need to renew my driving license, what should I do? Are there “guide dogs” that can notice hypoglycemia?
Tax exemptions	2	Will I get help to pay for the drugs? Do I have to pay for diabetes control tests?
Sport	7	Can I use sport supplements? What kind of physical activity can I do?
**Diet**	50	Are there any rules to follow for food consumption? Do meat and fish increase blood sugar?
**Prevention and complications**	25	How can I prevent diabetes? What tests do I need to take?

For each sub-theme, two questions are displayed as examples. The *Diet* and *Prevention and complications* themes do not have any sub-themes. The questions have been translated from Italian to English for the purpose of illustration

The first KB thus created contained roughly 120 questions, each with its own answer. The final KB shown here was expanded by including suggestions and comments from other doctors that tested AIDA while still in development, resulting in a total of 170 questions. The agent available on the web page and on Telegram both refer to this KB, while the voice application, available on the Amazon Alexa Skill platform, has its own content.

### AIDA Cookbot’s KB

One of the main subjects of interest that emerged from the interviews and the workshops is the diet theme. Food consumption is one of the major preoccupations for people with diabetes of both type 1 and type 2 [58]. For this reason, the scientific board decided to base the voice agent around food education by creating a system that provides recipes with a low glycemic index. The recipes were created by the medical personnel, thus ensuring the content’s reliability [14]. AIDA Cookbot’s KB consists of 61 recipes distributed in four categories: starters, first courses (pasta, rice), second courses (meat, fish) and desserts. While the board provided the recipes, the linguists on the team designed the interaction following the principles of voice-based human-machine interaction. The interaction is agent-driven and follows a series of steps:

- Step 1—The agent asks users if they have any allergies. This information is stored and used to exclude recipes that contain those allergens.
- Step 2—The agent asks what users would like to cook. No restrictions are imposed on the input: people might say an ingredient (e.g. *I would like to cook some rice*), a type of course (*Can you recommend me a first course?*), or a characteristic they would like (*I need a vegetarian recipe*, *What about something easy to cook?*).
- Step 3—The agent proposes a recipe based on users’ preferences. Users can accept it or ask for a different recipe. Once users find the recipe they want, the agent will proceed to list the ingredients. Diabetic patients can then proceed to the various steps of the recipe procedure, or hear the nutritional values. This latter information was deemed particularly important, given the dietary constraints imposed by the pathology. As the recipe is followed, users may ask to jump to the next step, to repeat the previous step or start again from the beginning.
- Unrestrained step—At any time, users may interrupt the flow by asking a general question about the Diet theme (e.g. *Am I allowed to eat candy?*. The diet theme questions present in AIDA Chatbot were indeed included in AIDA Cookbot in order to obtain a certain homogeneity between the two agents. Once the agent has answered the questions, it will prompt the user to come back to the main dialogue flow.

**Fig. 3:**
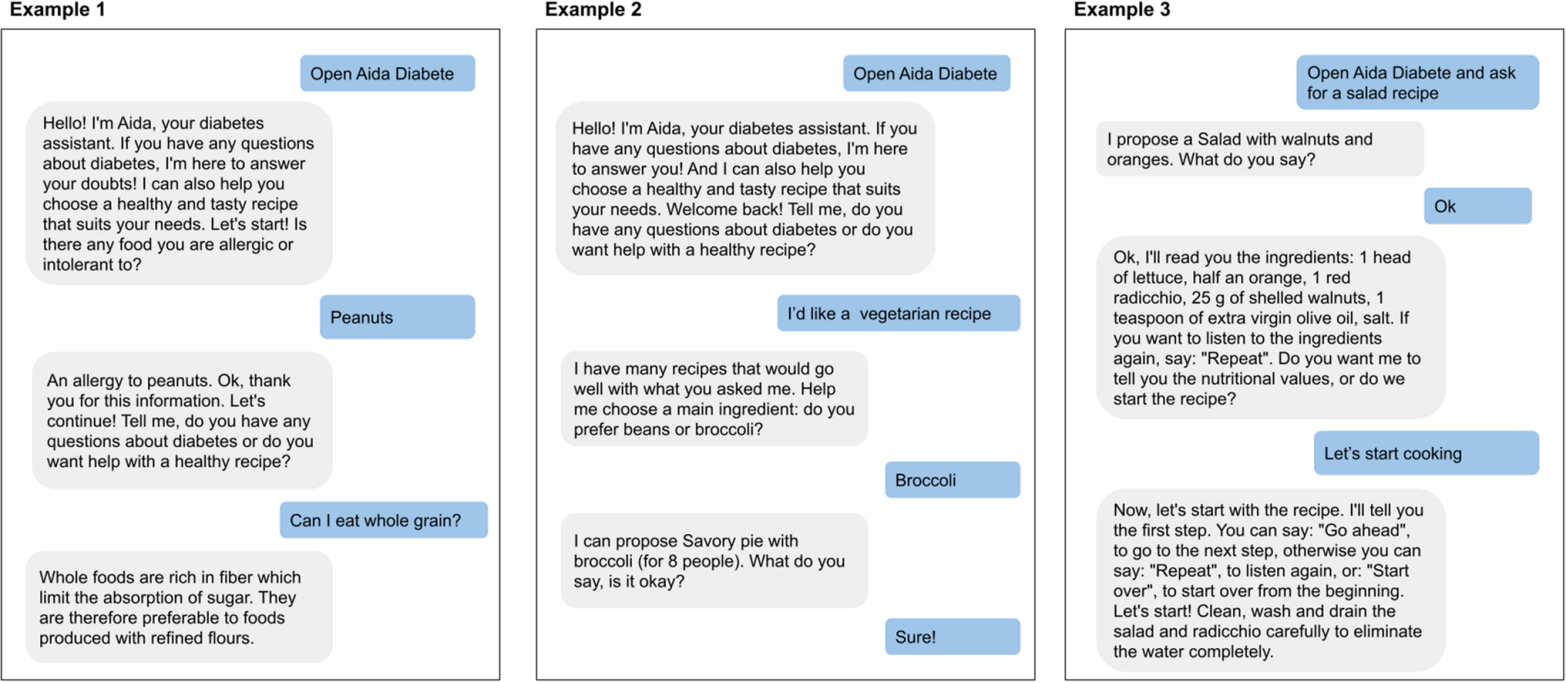
In Example 1, the user interacts with AIDA Cookbot for the first time and the agent asks for any allergy, in order to memorize it. Users can ask a Diet-related question or for a recipe. In Example 2, the user returns for a second interaction and expresses a preference for a recipe. AIDA guides the user towards something more specific. In Example 3, the user asks directly for a recipe and AIDA immediately proposes something related to the request

Figure 3 shows some example dialogue with AIDA Cookbot. AIDA Cookbot is delivered as an Alexa Skill integrated with proprietary software for the interaction model component. More detail about the Skill’s architecture can be found in Sect. 5. The agents are made available for free on a website,^2^ on Telegram^3^ and on the Amazon Alexa Skill store.^4^

## Conversational agents’ architecture

Both AIDA Chatbot and AIDA Cookbot were created by the same team from CELI company exploiting internal resources and proprietary software called Sophia Chatbot described in [59–61]. However, in order to create a voice-based experience, it was necessary to integrate the original software with one of the available platforms for building voice applications, Amazon Alexa. Therefore the two projects share some components but their architecture differs, according to their functioning and their endpoint. Specifically, AIDA Cookbot includes a connection with the Lex platform as well as the integration of a proprietary search engine.

### AIDA Chatbot’s architecture

**Fig. 4:**
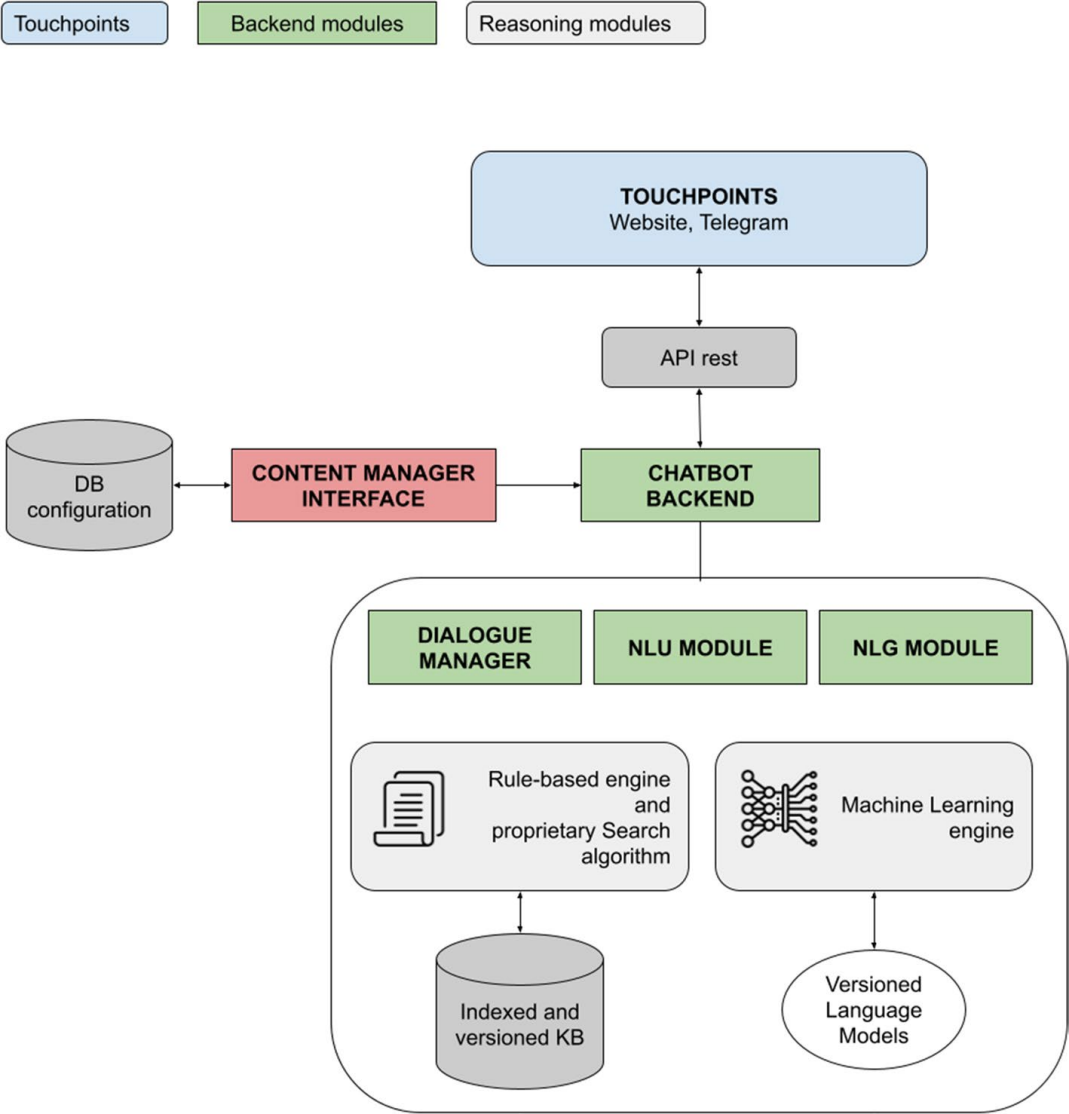
AIDA Chatbot architecture. The figure shows the interlacing between the touch points and the backend modules: the dialogue manager, the NLU and NLG modules, the reasoning ones (AIDA relies on a machine learning system as well as a rule-based engine). The communication with the touch points is established via API rest, while the contents of the agents themselves are managed in a separate database, through a specific content manager graphical interface

The architecture of the chatbot is composed of various modules that are integrated in a complete service. The backend includes the functionalities of Natural Language Understanding, Natural Language Generation and Dialogue Manager. The NLU module aims to understand the user input. The understanding operation can be defined as a classification task; details about the NLU module can be found in the next section. For architectural description purposes, it is sufficient to say that the NLU module classifies the user’s input according to its internal KB (described in the previous section). The NLG module is in charge of producing an answer to the question asked by the user. It either retrieves the appropriate response from a predefined set, or it can generate it dynamically from a template (e.g. in the “welcome” message, a variable is filled according to the time of day with “Good morning”, “Good afternoon” or “Good evening”). Figure 4 illustrates the whole architecture.

The Dialogue Manager is in charge of managing the conversation, making sure that the response is appropriate in the specific context and that the overall exchange is consistent. These three modules constitute the backbone of the chatbot back-end. They exploit both machine learning resources and rule-based approaches in order to build a reasoning system that is robust, but also refinable and explainable [62]. The following section will also illustrate the interplay between the rule-based engine and the machine learning engine. While the back end constitutes the logic of the application, the content is managed through the Content Manager Interface (CMI). In the CMI, linguists and product owners can insert new questions, modify the chatbot’s answers, enhance its small-talk capabilities, and many other operations to change the system’s content and improve its performance.

The separation of the content from the logic itself allows for flexible management of the experience. Any change in the KB, in the linguistic components, the re-indexing of the content or new language model training can be carried out autonomously, without interacting directly with the codebase.

### AIDA Cookbot’s architecture

**Fig. 5:**
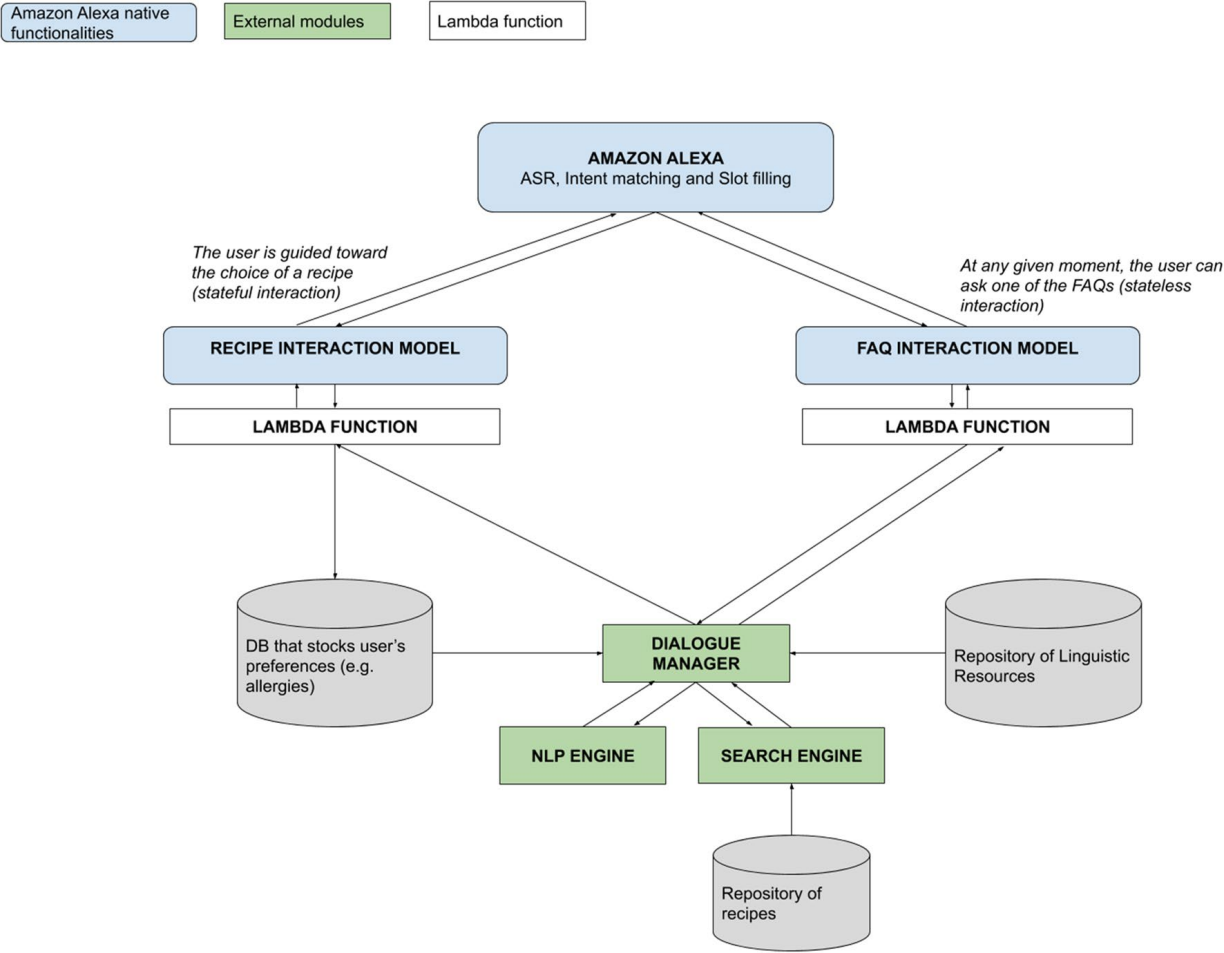
AIDA Cookbot’s architecture. The direction of the arrows symbolizes the flow of the information: from the Alexa endpoint towards one of the two models, down to the understanding engines. Once the proper answer has been crafted, it is passed up again through the up-pointing arrows

The architecture of the voice-based agent integrates different modules, as illustrated in Fig. 5. First of all, the Amazon Alexa intent recognizer engine matches the user input to one of the intents in the system. The intent recognizer is one of the native functions of Alexa that has to be employed, since it is the module in charge of listening and transcribing the words of the users. Once the sentences have been transcribed, they are matched to one of the intents configured in the Alexa console. AIDA is equipped with two kinds of intents: those that are part of the Recipe interaction model recognize a declaration of allergy or a request for a recipe; those that are part of the FAQ interaction model match user requests to one of the diet theme questions inserted in the agent. Various intents gather the necessary information through the Interaction model: allergens, main ingredient, course, etc. The matched intents and the slots are passed over to the proprietary NLP engine by means of the Lambda function.^5^

The native Lambda function sends the information about the matched intent, as well as eventual slots, to the correct part of the back end. The proprietary back end, composed of the Dialogue Manager and its sub-components, is in charge of directing the whole conversation and retrieving the appropriate content from the repositories. The Dialogue Manager, coupled with the NLP engine, orchestrates the conversation and decides what action is to be taken next in order to keep the dialogue consistent. The NLP engine analyzes the linguistic input it has available (the matched intent and its eventual slots) and extracts meaningful information through its own Named Entity Recognizer module. If the only input is the intent (with or without filled slots), the engine retrieves the answer from the repository of resources and passes it to the previous module. Otherwise, it will pass the information to the Search Engine, which will look for an appropriate recipe in its database, in accordance with the preferences expressed by the user. Once the engine retrieves the correct response, it passes it back to Alexa which will deliver it through its text-to-speech native module.

Similarly to the AIDA Chatbot architecture, all the repositories of linguistic resources or content (list of possible allergens and ingredients, database of recipes) are separated from the codebase. Its logic is agnostic to the resources it exploits, making it easier to update them at any given time.

## Natural language understanding and dialogue manager modules

This section shows the core algorithms that allow the chatbot to understand patients’ requests, provide the correct answer and manage the dialogue. While for AIDA Chatbot the understanding module is handled autonomously by the authors’ own software, for AIDA Cookbot the intent recognition is regulated solely by Amazon Alexa.

### AIDA Chatbot’s core system

Once the Knowledge Base has been drafted by the scientific board, all the contents are transferred into the CMI in order to be processed. Each question constitutes one of the classes (or *label*) that the NLU module will have to predict in order to give the correct answer.

#### Classification task with BERT

For the machine learning component (ML), each label must be enriched with variations of that question in order to provide the neural model with data. Those variations, also named expansions, are created manually by expert linguists. For a question like *Is it okay to eat fried food?*, the variations could be *I like fried food, can I eat it everyday?* or *Is it true that fried food is bad for people with diabetes?*. The goal is to extend the language model with different examples of how that question could be expressed.

The various examples of a label will be split between train and test. For each question 15 different expansions were generated. This quantity was determined empirically: we observed that for the current amount of questions in the KB (170 units), 15 expansions already accounted for high performances on the test set (0.8 F1 score or higher). This is due to the fact that each example is handcrafted by domain experts. The apparent scarcity of data is therefore compensated by its quality.

##### Model training

We use a bi-directional transformer [63] that has been pre-trained with the Italian language, known as BERT language model [64]. BERT produces several models through the various training cycles. If the selected model performs with an accuracy above a threshold of 0.8 in F1 score, it is implemented in the chatbot. Otherwise, it is discarded and the previous version is reinstated. This automated check enables a high quality baseline for the chatbot performances.

The model is trained for 5 epochs with a batch size of 16. These values are kept unmodified during the various training attempts in order to maintain consistency. The results are measured in terms of accuracy. The Appendix document details single labels performances for the model that was released into production.

##### Input processing

Once users write their questions, the sentences are processed through the proprietary NLP pipeline which includes normalization, tokenization, lemmatization, POS tagging, disambiguation and dependency parsing [61]. This pre-processed data is passed to the language model and it allows for better performance compared to using only the ML component [65]. BERT has yielded good results for classification tasks in different contexts [66–68], although those performances are usually obtained by pretraining the model [60, 69]. In this context, no specific pretraining was carried out, since the raw performance was already up to 0.8 in terms of accuracy. Such good results are due mainly to the good quality of entry data that was manually created and configured by expert linguists. Our methodology confirms the importance of data quality over quantity for ML applications [70].

If BERT fails to match the user input to one of the classes with sufficient confidence, a fallback mechanism makes another attempt at understanding the question. This fallback system is based on a proprietary rule-based engine.

#### Fallback classification with the rule-based engine

The rule-based engine serves multiple purposes: first, it enriches the input with additional information (e.g. specific synonyms of a term) before passing it to the language model. It also checks the user input against a series of patterns, in order to provide a quicker and simpler answer without consulting the neural part of the application. For instance, sentences such as “hello”, “goodbye”, “thank you” do not need to be classified, because they are quite unambiguous and there’s no need to employ a complex system to answer “see you soon”, “you’re welcome”. Moreover, this engine takes care of requests in the form of commands, such as “help” or “menu”. These inquiries directly trigger the Menu of contents, i.e. the list of the chatbot’s themes and sub-themes, without further linguistic analysis.

Secondly, the rule-based engine works as a fallback option in case the language model were to fail. The machine learning module enables a strong and automatized understanding of natural language; nonetheless, neural approaches have their limitations, and lack of explainability is one of them. Even though some work has been done in this direction [71, 72], in the setting of an industrial live chatbot the system still needs an explainable backup plan. If BERT is unable to classify the input with sufficient confidence, the chatbot relies on a proprietary algorithm based on Apache Lucene to look for meaningful keywords that were previously configured through the CMI.^6^ The threshold is set at accuracy = 0.4; below that value, the fallback system is activated.

The proprietary algorithm also handles the internal cohesion of the conversation. The dialogue with the chatbot is voluntarily stateless: in a user-driven interaction, the person may ask a different question or change the subject at any time. On the other hand, people naturally employ conversational strategies such as anaphoric references and the machine should be able to interpret even elliptic sentences [73, 74]. For this reason, the algorithm also works as a light form of Dialogue Manager, keeping track of the topic that is under discussion. Its topic memorization ability empowers the resolution of these kinds of dialogues:

USER: I was just diagnosed. Does the therapy prescribe mandatory insulin injections?

CHATBOT: Injection therapies can be a cause of concern, but it depends on your diagnosis. In type 1 diabetes, therapy is necessarily injective, while in type 2 diabetes there are both injection and oral therapies.

USER: And do *they* hurt?

CHATBOT: I understand your concern about the pain of insulin injections - I can reassure you that they are not particularly painful, as small needles (5-6 mm) are used. [...]

In this example, a user employs the anaphoric pronoun *they*. The chatbot is able to resolve the missing information by remembering the topic from the previous question, i.e. “insulin injections”. Details about the algorithm in charge of the anaphora resolution are described in the work by Bianchini et al. [59].

### AIDA Cookbot’s interaction model

As has been stated before, the NLU module for the voice-based agent is managed directly by Amazon technology and therefore little work can be done on that by a third party [75]. On the other hand, the interaction model—i.e. the Skill’s dialogue manager module—has been implemented in-house, in order to have complete control over the conversational path.

**Fig. 6:**
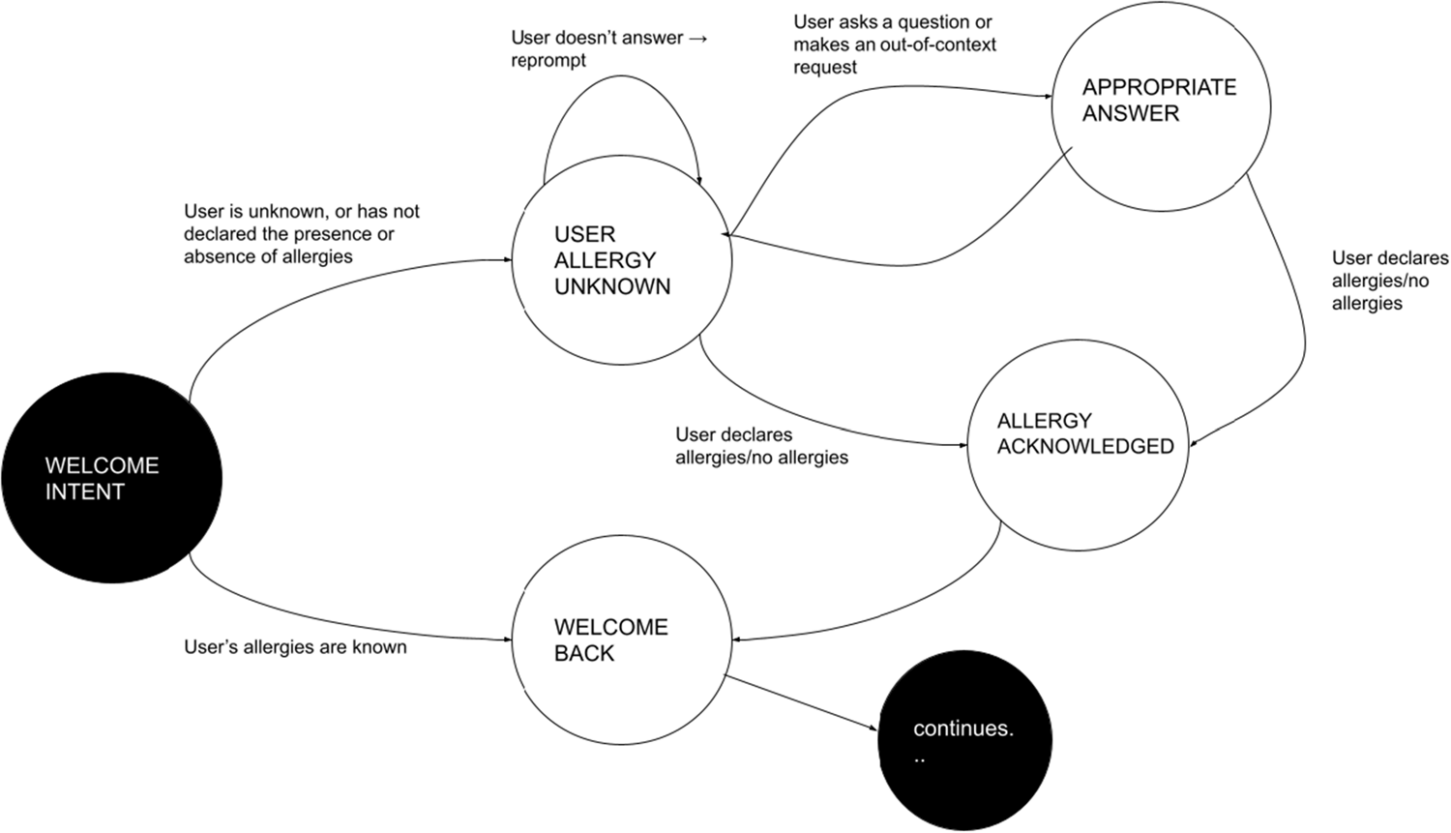
The initial states of AIDA Cookbot’s interaction model

AIDA Cookbot’s interaction model relies on a finite state automaton (FSA) structure. Although FSAs have their limitations [76], it was deemed to be the best strategy in this context where developers have limited control over the NLU part. Given that the input information is already pre-processed by Alexa and therefore somehow implicit, an FSA enables control of the actions of the dialogue system by implementing a stateful interaction. Figure 6 shows the first part of the automaton. When a user enters the Skill, the system checks against its database whether the presence or absence of allergies has already been stored. If the user’s preferences are known, AIDA produces a welcome back message and proceeds to the rest of the dialogue. Otherwise, the Skill asks if the person has any allergies. The user can answer or ask one of the questions related to the diet theme (or produce an out-of-context request; however, this possibility is always true). In any case the system will retrieve the appropriate answer and then go back to the user allergy unknown state.

## Release and usage data

The medical board, the facilitators and the computational linguists began the design process of both AIDAs in February 2020. Shortly after Italy was placed in lockdown due to the COVID-19 situation, the project was strengthened by the increasing needs of patients who could no longer confer with their doctors in person. Both AIDA Chatbot and AIDA Cookbot were made available to the public from July 2020 and officially launched with the communication campaign on October 12, 2020. Between July and October, the systems were refined by incorporating the interaction with medical personnel invited to test the agents. Afterwards, in order to promote the existence of AIDAs, a digital advertising strategy was put in place.

The main objective of this action was to expand the visibility of the Chatbot and the Cookbot to the general public, generate interest and boost traffic to the dedicated landing page. Given the high technological component of the project, we were unsure that users would agree to engage with this tool.

The controlled launch and promotion of the agents also helped to set expectations around the systems [77]. All editorials stressed the recent nature of the agents, the fact that they were machines (and therefore should not be expected to behave as their doctors) and outlined the contents the CAs were able to deliver.

### AIDA Chatbot usage data and performance

Table 2 presents usage data from the months of November and December 2020. Since some users may interact with the agent during multiple sessions, the number of unique users shall always be lower than the number of sessions. The number of requests coincides with the number of questions posed by the users; however, some of them may be commands (e.g. “menu”) and not natural language sentences. The number of questions posed in natural language is marked in the *Assessable requests* line. The mean duration of a session is 70 s: even though it is possible to argue that the goal of a chatbot is to engage in dialogue with a person for the longest time possible, AIDA is a task-oriented agent. Users should be able to get the information they are seeking in the shortest possible time; therefore we argue that the data fits the scenario of a user who is looking for specific information and gets it with virtually no latency. The majority of the users come from the website-based AIDA, while a minority access the agent through Telegram. This behavior can be explained with the fact that it is easier or preferable to reach a web page via a link or a plain URL rather than download a specific app.

**Table 2:** AIDA Chatbot usage data from November 1st, 2020 to December 30th, 2020

KPIs	Values
Unique users	3788
Sessions	3960
Number of requests	11,806
Assessable requests	7668
*Theme: Diet*	2037
*Theme: Diagnosis → General information*	1200
*Theme: Diagnosis → Symptoms of diabetes*	576
Average session duration	70 s

The most asked subject is *Diet*, the *Diagnosis* → *General information* theme is in second place, while *Diagnosis* → *Symptoms of diabetes* is placed third. This user behavior is consistent with the design premises of AIDAs that anticipated the need for two different systems: one written agent with different information spanning the whole spectrum of diabetes, and a voice-based agent focused on the diet theme that provides recipes that comply with diabetes requirements.

#### Validation

Even though the system is equipped with a fallback system, the rule-based algorithm only activated on 32% of the total number of requests. This means that for most of the questions posed by the users, the BERT-based engine was confident enough to provide the answer. Unfortunately it is not easy to compare our model’s performance with standard baselines [78, 79], because the text upon which BERT performs its prediction has already been pre-processed by a specific pipeline that modifies the original input [61]. In order to shed some light into the actual performances of AIDA Chatbot with real users, we conducted a manual validation task. This task aims to evaluate the performance of the text-based agent through a human-in-the-loop approach [80].

It is carried out by an annotator with a formal training in linguistics. This person also took part in the co-design phase of the project, so that he was already aware of the content of the system and he was also privy to the internal functioning of the agent. His knowledge was necessary to interpret the quality of the questions and the correctness of the given answer. The annotator can mark any answer given by the chatbot as correct, error, out of context, not applicable or unknown theme. The out of context tag is used when a user makes a request that is beyond the agent’s scope; non applicable is used for commands or other input that is not useful to evaluate; unknown theme marks a question that is within the theme of diabetes, but it is not currently included in the agent’s KB. Unknown theme questions can then be analysed by the scientific board and, if they are deemed appropriate, can be included in the KB along with their answer. When an answer is marked as an error, the annotator can select the correct answer that should have been provided for that request. The system will then integrate the human feedback into its dialogue policy and learn the correct behavior for that occasion. The next time a user asks a similar question, the agent will know the right answer, thanks to the instruction given during the validation session.

The Validation task was performed on a portion of the data for November. Table 3 shows the number of questions that were evaluated together with the evaluation results. The number of correct answers is the highest (42% of the total assessed requests), while the errors amount to 31%. This is due to the fact that while asking the questions, real users may employ linguistic structures that are rather different to the ones drafted by the scientific board. For instance, many users often employed the term “blood glucose” as a synonym of diabetes, even though it is not strictly correct. Other users misspelled certain technical terms such as “glycated hemoglobin”. The confusion and the incorrect writing of medical terms resulted in the match of wrong answers by the system. It is also interesting to note the quantity of unknown theme questions (13%): naturally, users will ask about subjects that were not included in the KB initially. A lot of questions were concerned with the possibility to eat specific food such as pizza, risotto, or *polenta* (typical Italian cornmeal) that were not taken into account in the construction of the KB. Out of context questions (14%) cover requests that were not pertinent with the scope of the agent: trolling, insults and such.

**Table 3:** AIDA Chatbot performance metrics from November 1st, 2020 to November 30th, 2020

Performance metrics	Values
Number of requests	10,495
Assessable requests	6860
Assessed requests	941
Correct	394
Error	291
Out of context	133
Unknown theme	123

This analysis serves two purposes: on the one hand, it improves the accuracy of the agents through the human-in the-loop approach. On the other hand, it discovers untouched themes that users find interesting and that can later be incorporated into the system’s knowledge.

### AIDA Cookbot usage data and performances

Table 4 presents usage data from the months of November and December 2020. The lower number of users for the cookbot compared to the chatbot can be explained by the fact that in order to access AIDA Cookbot, people should possess an Amazon Alexa enabled device. Commercial research conducted in 2019 demonstrated that the prevalence of Alexa devices in Italy is still quite low compared to other countries.^7^ It is also true that it is possible to use Alexa’s features from a mobile app, but it is not a widespread phenomenon. The mean duration of the session is higher, as is to be expected from voice-based interaction.

**Table 4:** AIDA Cookbot usage data from November 1st, 2020 to December 30th, 2020

KPIs	Values
Unique users	640
Sessions	488
Number of requests	2920
Assessable requests	7668
Average session duration	89 s

Unfortunately, it is not possible to provide information about users’ requests with the same granularity as AIDA Chatbot. The Alexa Skill console does provide aggregated analytics about the skill’s usage: for instance, intent recognition confidence is estimated at 80% and endpoint latency at less than 292.8 ms. However, without knowing the exact sentences pronounced by the user, it is hard to interpret whether the amount of “NoIntent” matches corresponds to actual “no” requests. Moreover, AIDA Cookbot is mainly structured as a stateful interaction, since its goal is to provide guided recipes based on a series of preferences; nonetheless, users may ask one of the diet related questions at any time, going out of the interaction path momentarily, only to return to it after a while. Analytics may then show a deviation from the mainstream path, but without knowing what was the question asked by the user, it is difficult to tell if it was an out-of-context request, an unknown topic, or simply a question related to the recipe.

## Discussion

The goal of our work was threefold: to apply typical HCI techniques to a collaborative design of two different CAs, one text-based and one voice-based; to demonstrate the effectiveness of a complex architecture that relies on both machine learning and rule-based engines; and finally, to test the agents *in the wild*. Specifically, we aimed to investigate whether Italian users would accept such a technology and how would they use it.

With regard to the first objective, our design approach allowed for a quick development of the agents: the workshops gathered all the necessary requirements of the stakeholders and served as constant guidelines during the whole process. By exploiting this methodology, we were constantly checking the progress against the initial specifications, thus ensuring a final product compatible with the needs of patients and clinicians.

The results shown in the previous section demonstrated the success of the second goal. The classification system composed by BERT language model together with a proprietary algorithm was able to classify 91% of the requests with sufficient confidence. We carried out a validation task to gain more insights into the system’s performance: by means of a manual analysis we observed that in the majority of occurrences the agent correctly interpreted users’ requests. In the cases where the CA did not output the right answer, the annotator was able to provide feedback to the agent by indicating the correct label. This human-in-the-loop approach aims to improve the agent’s performance by embedding the annotator’s feedback into the classification task. In the future, we expect to see a reduction in the number of error occurrences thanks to a constant monitoring of the interactions.

Finally, we tackled the third objective by releasing both AIDAs to the general public on widely accessible platforms. Together with the digital advertising campaign, these actions aimed to attract as many users as possible. We believe that the usage data demonstrates the success of these activities: considering the fact that both AIDAs talk in Italian (therefore they have a limited public) and they are dedicated to a subset of the general population (those affected with diabetes), we reached almost 4000 unique users in the span of 2 months. Unfortunately, this kind of data about industrial-based systems in production is usually not available and it is therefore hard to make a comparison with other CAs. Through the analysis of the dialogues we confirmed some expected behavior (e.g. the importance of diet related questions), while others were unforeseen, such as the limited interest in the sport theme. We were also able to gather new questions that are not currently supported but are indeed relevant to the agent’s scope. The 133 occurrences marked as unknown theme constitute excellent suggestions for future expansion of AIDAs’ KB.

## Conclusion

In this work we presented AIDA, the Artificial Intelligence Diabetes Assistant. AIDA bases its scientific premises on Therapeutic Education, a teaching system that proposes to train patients in order to increase their autonomy and enable them to reach a new level of well-being. The final objective of the project is to offer tools that can guide patient behavior until a balance is obtained between their needs and the constraints imposed by the pathology. In fact, Therapeutic Education is intended to make people with diabetes as conscious and autonomous as possible in the management of their condition [10]. In order to do so, AIDA was devised as two autonomous but related CAs: AIDA Chatbot and AIDA Cookbot.

AIDA Chatbot is based on written communication and is deployed through a website and via the chat application Telegram. It provides answers to a vast number of questions concerning various aspects of diabetes. AIDA Cookbot, on the other hand, focuses on a specific subject: diet-related questions and advice for people with diabetes. AIDA Cookbot is delivered as an Alexa Skill and therefore expresses itself as voice. It recommends healthy and savory recipes that have been approved by the scientific board. The article reports usage data from the month of November and December 2020. Since the sponsoring campaign started in October, those 2 months represent the most recent and most significant usage data for both agents. AIDAs are, to the best of our knowledge, the sole examples of CAs for diabetes patients that employ the Italian language.

While they were meant primarily for patients, they can be used by caregivers as well. Moreover, physicians may find them useful to help them educate newly diagnosed patients and the general public. For this reason both agents are made publicly available for free, with no restrictions and no technical requirements other than a device with an Internet connection. Different requirements by all the stakeholders were taken into account during the design phase: the subjects of interest for the patients, their preoccupations, and the diabetologists’ point of view.

The aim of this work was to demonstrate the feasibility of two different CAs for TE in the context of diabetes. While the data suggests that the public is willing to interact with those CAs, it would be useful to capture the opinions of users about them. In the future, we plan to conduct a precise evaluation of the systems with a controlled group of patients. It is our intention to investigate what benefits users will experience once they discover and use AIDAs, and whether the dialogues with CAs will have an impact on their clinical progression or psychological well-being.

## Supplementary Information

Below is the link to the electronic supplementary material.

Supplementary file1 (DOCX 15 KB)

## Data Availability

Both systems described in the project are publicly available. The dataset generated and analysed during the current study is not publicly available due to the fact that users may disclose personal information (even though such information is never elicited) to the agents that cannot be divulged. However, once data is accurately anonymized, it could be made available from the corresponding author on reasonable request.

## Footnotes

1 Alexa Skills are the voice app that can be experienced via Alexa-enabled devices and mobile interface provided by Amazon. For more information: https://developer.amazon.com/en-US/alexa/alexa-skills-kit.

2 https://www.aidachatbot.it/.

3 Handle: @aida_chatbot.

4 Provided that the specific Alexa device can access Italian content, it is sufficient to say *Alexa, apri Aida Diabete* in order to access the skill.

5 https://docs.aws.amazon.com/lambda/latest/dg/welcome.html.

6 https://lucene.apache.org/.

7 https://www.celi.it/blog/2019/08/assistenti-vocali-la-ricerca-di-celi-rivela-le-abitudini-degli-italiani/.

## Code availability

The systems described in the work are built upon proprietary software. In some cases, the software relies on publicly available resources (e.g. language models) or it is connected to other endpoints (e.g. Amazon Alexa). These instances are clearly specified in the text. Footnotes or bibliographical references allow readers to further explore the resources.
