# Supplementary material for: Diabetes and conversational agents: the AIDA project case study
Source: Discov Artif Intell. 2021 Sep 22;1(1):4. doi: 10.1007/s44163-021-00005-1 (PMC8456073; doi:10.1007/s44163-021-00005-1)
Supplement: Supplementary file 1 — Supplementary file1 (DOCX 15 KB) [file 44163_2021_5_MOESM1_ESM.docx]

**APPENDIX**

The following table reports the performance of all the labels in the training data in terms of F1 score, precision and recall. The data refers to the version of the model that was released in production and was in charge of interpreting users’ input at the time (November - December 2020).

The loss for the final step is 0.20 and the total training time amounts to 1 hour and 59 minutes. The global values are: accuracy = 0.84, F1 score = 0.82, precision = 0.85, recall = 0.84.

| **Label** | **F1 score** | **Precision** | **Recall** |
| --- | --- | --- | --- |
| 1 | 0.9714286 | 0.9444444 | 1.0 |
| 2 | 0.72727275 | 1.0 | 0.5714286 |
| 3 | 0.25 | 0.33333334 | 0.2 |
| 4 | 0.7368421 | 0.6363636 | 875 |
| 5 | 0.7058823 | 0.6666667 | 0.75 |
| 6 | 0.8 | 1.0 | 0.6666667 |
| 7 | 1.0 | 1.0 | 1.0 |
| 8 | 0.71428573 | 0.71428573 | 0.71428573 |
| 9 | 0.9285714 | 0.8666667 | 1.0 |
| 10 | 0.75 | 0.75 | 0.75 |
| 11 | 1.0 | 1.0 | 1.0 |
| 12 | 0.7692308 | 0.8333333 | 0.71428573 |
| 13 | 0.8235294 | 1.0 | 0.7 |
| 14 | 0.75000006 | 1.0 | 0.6 |
| 15 | 0.61538464 | 0.6666667 | 0.5714286 |
| 16 | 0.53846157 | 0.53846157 | 0.53846157 |
| 17 | 0.6666667 | 0.5 | 1.0 |
| 18 | 0.4 | 0.5 | 0.33333334 |
| 19 | 1.0 | 1.0 | 1.0 |
| 20 | 0.72 | 0.6 | 0.9 |
| 21 | 1.0 | 1.0 | 1.0 |
| 22 | 0.6666667 | 0.5 | 1.0 |
| 23 | 0.8888889 | 0.8 | 1.0 |
| 24 | 0.9090909 | 0.8333333 | 1.0 |
| 25 | 0.71428573 | 0.5555556 | 1.0 |
| 26 | 1.0 | 1.0 | 1.0 |
| 27 | 0.6666667 | 0.5 | 1.0 |
| 28 | 0.61538464 | 0.5714286 | 0.6666667 |
| 29 | 0.8571428 | 1.0 | 0.75 |
| 30 | 0.9090909 | 0.8333333 | 1.0 |
| 31 | 1.0 | 1.0 | 1.0 |
| 32 | 1.0 | 1.0 | 1.0 |
| 33 | 1.0 | 1.0 | 1.0 |
| 34 | 1.0 | 1.0 | 1.0 |
| 35 | 1.0 | 1.0 | 1.0 |
| 36 | 1.0 | 1.0 | 1.0 |
| 37 | 0.75 | 0.75 | 0.75 |
| 38 | 0.8888889 | 1.0 | 0.8 |
| 39 | 0.9090909 | 0.8333333 | 1.0 |
| 40 | 0.59999996 | 0.5 | 0.75 |
| 41 | 1.0 | 1.0 | 1.0 |
| 42 | 0.8 | 1.0 | 0.6666667 |
| 43 | 1.0 | 1.0 | 1.0 |
| 44 | 0.8 | 0.6666667 | 1.0 |
| 45 | 0.6666667 | 1.0 | 0.5 |
| 46 | 0.5 | 0.6666667 | 0.4 |
| 47 | 1.0 | 1.0 | 1.0 |
| 48 | 0.8888889 | 0.8 | 1.0 |
| 49 | 1.0 | 1.0 | 1.0 |
| 50 | 1.0 | 1.0 | 1.0 |
| 51 | 1.0 | 1.0 | 1.0 |
| 52 | 0.8888889 | 0.8 | 1.0 |
| 53 | 1.0 | 1.0 | 1.0 |
| 54 | 1.0 | 1.0 | 1.0 |
| 55 | 0.8888889 | 0.8 | 1.0 |
| 56 | 1.0 | 1.0 | 1.0 |
| 58 | 0.4 | 1.0 | 0.25 |
| 59 | 1.0 | 1.0 | 1.0 |
| 60 | 1.0 | 1.0 | 1.0 |
| 63 | 1.0 | 1.0 | 1.0 |
| 64 | 0.5 | 1.0 | 0.33333334 |
| 65 | 1.0 | 1.0 | 1.0 |
| 66 | 0.53333336 | 0.5 | 0.5714286 |
| 67 | 0.8571428 | 0.75 | 1.0 |
| 68 | 0.8888889 | 1.0 | 0.8 |
| 69 | 1.0 | 1.0 | 1.0 |
| 70 | 1.0 | 1.0 | 1.0 |
| 71 | 1.0 | 1.0 | 1.0 |
| 72 | 0.8 | 0.8 | 0.8 |
| 73 | 1.0 | 1.0 | 1.0 |
| 74 | 0.84210527 | 0.72727275 | 1.0 |
| 75 | 0.8 | 1.0 | 0.6666667 |
| 76 | 1.0 | 1.0 | 1.0 |
| 77 | 1.0 | 1.0 | 1.0 |
| 78 | 1.0 | 1.0 | 1.0 |
| 79 | 1.0 | 1.0 | 1.0 |
| 80 | 0.8 | 1.0 | 0.6666667 |
| 81 | 1.0 | 1.0 | 1.0 |
| 82 | 1.0 | 1.0 | 1.0 |
| 83 | 1.0 | 1.0 | 1.0 |
| 84 | 0.33333334 | 1.0 | 0.2 |
| 85 | 0.8 | 0.6666667 | 1.0 |
| 86 | 1.0 | 1.0 | 1.0 |
| 87 | 0.8571428 | 1.0 | 0.75 |
| 88 | 1.0 | 1.0 | 1.0 |
| 89 | 0.75 | 0.75 | 0.75 |
| 90 | 0.75000006 | 0.6 | 1.0 |
| 91 | 0.9090909 | 0.8333333 | 1.0 |
| 92 | 0.8 | 1.0 | 0.6666667 |
| 93 | 0.6666667 | 1.0 | 0.5 |
| 94 | 0.5 | 0.5 | 0.5 |
| 95 | 1.0 | 1.0 | 1.0 |
| 96 | 0.77777773 | 0.7 | 875 |
| 97 | 0.8888889 | 0.8 | 1.0 |
| 98 | 1.0 | 1.0 | 1.0 |
| 99 | 1.0 | 1.0 | 1.0 |
| 100 | 0.8888889 | 0.8 | 1.0 |
| 101 | 0.6666667 | 0.6 | 0.75 |
| 102 | 1.0 | 1.0 | 1.0 |
| 103 | 1.0 | 1.0 | 1.0 |
| 104 | 0.41666666 | 0.2631579 | 1.0 |
| 105 | 1.0 | 1.0 | 1.0 |
| 106 | 1.0 | 1.0 | 1.0 |
| 107 | 1.0 | 1.0 | 1.0 |
| 108 | 1.0 | 1.0 | 1.0 |
| 109 | 1.0 | 1.0 | 1.0 |
| 110 | 0.923077 | 0.85714287 | 1.0 |
| 111 | 1.0 | 1.0 | 1.0 |
| 112 | 1.0 | 1.0 | 1.0 |
| 113 | 0.75000006 | 0.6 | 1.0 |
| 114 | 1.0 | 1.0 | 1.0 |
| 115 | 0.9166666 | 0.9166667 | 0.9166667 |
| 116 | 0.8666667 | 0.7647059 | 1.0 |
| 117 | 0.75409836 | 0.88461536 | 0.6571429 |
| 118 | 0.5714286 | 0.6666667 | 0.5 |
| 119 | 1.0 | 1.0 | 1.0 |
| 120 | 1.0 | 1.0 | 1.0 |
| 121 | 1.0 | 1.0 | 1.0 |
| 122 | 1.0 | 1.0 | 1.0 |
| 123 | 1.0 | 1.0 | 1.0 |
| 124 | 0.8571428 | 0.75 | 1.0 |
| 125 | 1.0 | 1.0 | 1.0 |
| 126 | 1.0 | 1.0 | 1.0 |
| 127 | 1.0 | 1.0 | 1.0 |
| 128 | 1.0 | 1.0 | 1.0 |
| 129 | 0.8 | 0.6666667 | 1.0 |
| 130 | 1.0 | 1.0 | 1.0 |
| 131 | 0.8888889 | 0.8 | 1.0 |
| 132 | 1.0 | 1.0 | 1.0 |
| 133 | 1.0 | 1.0 | 1.0 |
| 134 | 0.9090909 | 0.8333333 | 1.0 |
| 135 | 1.0 | 1.0 | 1.0 |
| 136 | 1.0 | 1.0 | 1.0 |
| 137 | 1.0 | 1.0 | 1.0 |
| 138 | 1.0 | 1.0 | 1.0 |
| 139 | 1.0 | 1.0 | 1.0 |
| 140 | 1.0 | 1.0 | 1.0 |
| 141 | 1.0 | 1.0 | 1.0 |
| 142 | 1.0 | 1.0 | 1.0 |
| 143 | 0.86956525 | 0.7692308 | 1.0 |
| 144 | 0.8571428 | 1.0 | 0.75 |
| 145 | 0.6666667 | 0.5714286 | 0.8 |
| 146 | 0.45 | 0.8181818 | 0.31034482 |
| 147 | 1.0 | 1.0 | 1.0 |
| 148 | 0.8888889 | 1.0 | 0.8 |
| 149 | 0.9411765 | 0.8888889 | 1.0 |
| 150 | 1.0 | 1.0 | 1.0 |
| 151 | 0.8571428 | 0.75 | 1.0 |
| 152 | 1.0 | 1.0 | 1.0 |
| 153 | 1.0 | 1.0 | 1.0 |
| 154 | 1.0 | 1.0 | 1.0 |
| 155 | 0.8571428 | 1.0 | 0.75 |
| 156 | 1.0 | 1.0 | 1.0 |
| 157 | 0.8 | 1.0 | 0.6666667 |
| 158 | 0.44444445 | 0.5 | 0.4 |
| 159 | 0.8571428 | 1.0 | 0.75 |
| 160 | 1.0 | 1.0 | 1.0 |
| 161 | 0.8 | 1.0 | 0.6666667 |
| 162 | 1.0 | 1.0 | 1.0 |
| 163 | 1.0 | 1.0 | 1.0 |
| 164 | 1.0 | 1.0 | 1.0 |
| 165 | 0.8571428 | 1.0 | 0.75 |
| 166 | 1.0 | 1.0 | 1.0 |
| 167 | 1.0 | 1.0 | 1.0 |
| 168 | 0.8888889 | 0.8 | 1.0 |
| 169 | 1.0 | 1.0 | 1.0 |
| 170 | 1.0 | 1.0 | 1.0 |
| 171 | 0.8571428 | 1.0 | 0.75 |
| 172 | 0.8888889 | 0.8 | 1.0 |
| 173 | 0.8333333 | 0.71428573 | 1.0 |
